# Knowledge and attitude towards cutaneous leishmaniasis among rural endemic communities in Shara’b district, Taiz, southwestern Yemen

**DOI:** 10.1186/s12879-021-05965-4

**Published:** 2021-03-17

**Authors:** Talal H. Alharazi, Najoua Haouas, Hesham M. Al-Mekhlafi

**Affiliations:** 1 Department of Clinical Laboratory Sciences, College of Applied Medical Sciences, University of Hail, Hail, Kingdom of Saudi Arabia; 2 Department of Microbiology and Immunology, Faculty of Medicine and Health Sciences, Taiz University, Taiz, Yemen; 3 Laboratory of Medical and Molecular Parasitology-Mycology LP3M (code LR12ES08), Department of Clinical Biology B, Faculty of Pharmacy, University of Monastir, Monastir, Tunisia; 4 Medical Research Centre, Jazan University, Jazan, Kingdom of Saudi Arabia; 5 Department of Parasitology, Faculty of Medicine, University of Malaya, 50603 Kuala Lumpur, Malaysia; 6 Department of Parasitology, Faculty of Medicine and Health Sciences, Sana’a University, Sana’a, Yemen

**Keywords:** Cutaneous leishmaniasis, Neglected tropical diseases, Infectious diseases, Knowledge, Attitude, Yemen

## Abstract

**Background:**

Cutaneous leishmaniasis (CL), a neglected tropical disease, represents a significant public health problem in many endemic countries including Yemen. The ongoing armed conflict that started in March 2015 has had a negative impact on the entire healthcare system as well as on infectious disease control programmes. Therefore, this cross-sectional study aimed to assess knowledge and attitude towards CL among rural endemic communities in southwestern Yemen.

**Methods:**

Five hundred households in five areas of Shara’b district of Taiz governorate were randomly selected to participate in a quantitative survey. A pretested structured questionnaire was used to collect data on the sociodemographic characteristics of the participants, their knowledge and attitude towards CL and their knowledge on the sand fly vector.

**Results:**

The analysis was conducted on a final sample of 466 individuals (62.7% males and 37.3% females) aged between 18 and 70 years. Among the participants, 21.5% were non-educated while 39.7 and 20.8% had completed secondary school and tertiary education, respectively. Although the participants were aware of CL, about three quarters (77.7%) of them had poor overall knowledge about disease transmission, clinical presentation, treatment, and prevention. Interestingly, approximately half of the participants (49.1%) were able to differentiate sand flies from other flies and mosquitoes. However, only 14.8% of the participants knew about the role of the phlebotomine sand fly in the transmission of CL. Only 36.6% believed that CL can be prevented and 49.6% had a negative attitude towards the disease. Univariate and multivariate analyses showed that age and gender were the significant determinants of knowledge about CL and the sand fly vector among the studied population.

**Conclusion:**

A poor level of knowledge about the different epidemiological aspects of CL was found among rural CL-endemic communities in Taiz. This factor, together with the major collapse of the healthcare infrastructure due to the ongoing civil war in Yemen, may be contributing to the continued endemicity of CL in the governorate. It is therefore recommended that health education on CL transmission and prevention should be provided to the targeted communities.

**Supplementary Information:**

The online version contains supplementary material available at 10.1186/s12879-021-05965-4.

## Background

Cutaneous leishmaniasis (CL) is a vector-borne infectious disease caused by the haemoflagellate protozoan of *Leishmania* (*L*.), a genus of the *Trypanosomatidae* family. It is transmitted via the bite of infected female phlebotomine sand flies. The main *Leishmania* species implicated in CL are *L. major, L. tropica* and *L. aethiopica* [1]. Incidences of CL occur in more than 89 countries and three territories in five continents, and are particularly prevalent in the tropics and sub-tropics [2]. Globally, it is estimated that there are 750,000 to one million new cases annually, and that 350 million people are at risk of infection from all CL forms [2, 3]. Cutaneous leishmaniasis can mainly be found around the Mediterranean basin, in the Middle East, the Horn of Africa, and the Indian subcontinent [3]. Clinically, CL is a spectral and extremely stigmatising disease that predominantly affects the face and exposed parts of the body. It usually presents as single or multiple nodules and ulcers on the skin [1]. Although CL is generally not fatal, clinical symptoms can lead to serious disfiguring scars that lead to social stigmatisation and psychological suffering as well as financial loss [4]. Cutaneous leishmaniasis is considered a neglected tropical disease as policymakers and public health professionals are not making enough effort to control its prevalence [3].

In Yemen, although the first report of CL was documented in 1933 [5], and the disease still accounts for approximately 5% of skin diseases [6], little is known about the epidemiological features and temporospatial evolution of the disease. Previous studies have revealed that CL is mainly endemic in the northwestern, southwestern, and central highland areas of the country [7–10]. According to the World Health Organisation (WHO), 4763 CL cases were reported in Yemen in 2018 [11]. The main causative species is *L. tropica*, with *Phlebotomus arabicus* implicated as the potential vector [12] and rock hyrax as the reservoir host [7]. Hajjah and Al-Bayda governorates in the northwestern and central highlands, respectively, have been found to have the highest number of reported cases [6, 7] while Lahj and Taiz governorates have been reported to have the highest number of reported cases in the southwestern region [8–10].

The ongoing civil war in Yemen, which began in 2015, has resulted in the deterioration of the healthcare infrastructure, a breakdown in the delivery of control programmes, the collapse of the healthcare system, and a shortage in the healthcare workforce, and has also impeded access by the population to healthcare facilities [13]. Consequently, the incidence of CL has increased rapidly, and new foci of transmission have also been reported, especially in the northwestern region [7]. Consequently, in the absence of a robust healthcare infrastructure and effective surveillance system, urgent action is required to curtail the incidence and spread of this infectious disease.

Many studies have revealed that an effective way of tackling infectious diseases is to improve community knowledge and attitude because these attributes play an important role in the prevention and control of such diseases. Although numerous studies have been carried out in different endemic regions worldwide in order to assess knowledge about and attitude towards CL among various populations, to the best of our knowledge no studies have been conducted in Yemen. Therefore, the aim of the current study was to evaluate the knowledge about and attitude towards CL among rural communities in five CL-endemic areas of Shara’b district in Taiz governorate, southwestern Yemen. It is hoped that the data obtained by this study will provide valuable information that can be utilised in the development of an effective control strategy to eliminate the transmission of this neglected tropical disease in rural Yemen.

## Materials and methods

### Study area

**Fig. 1:**
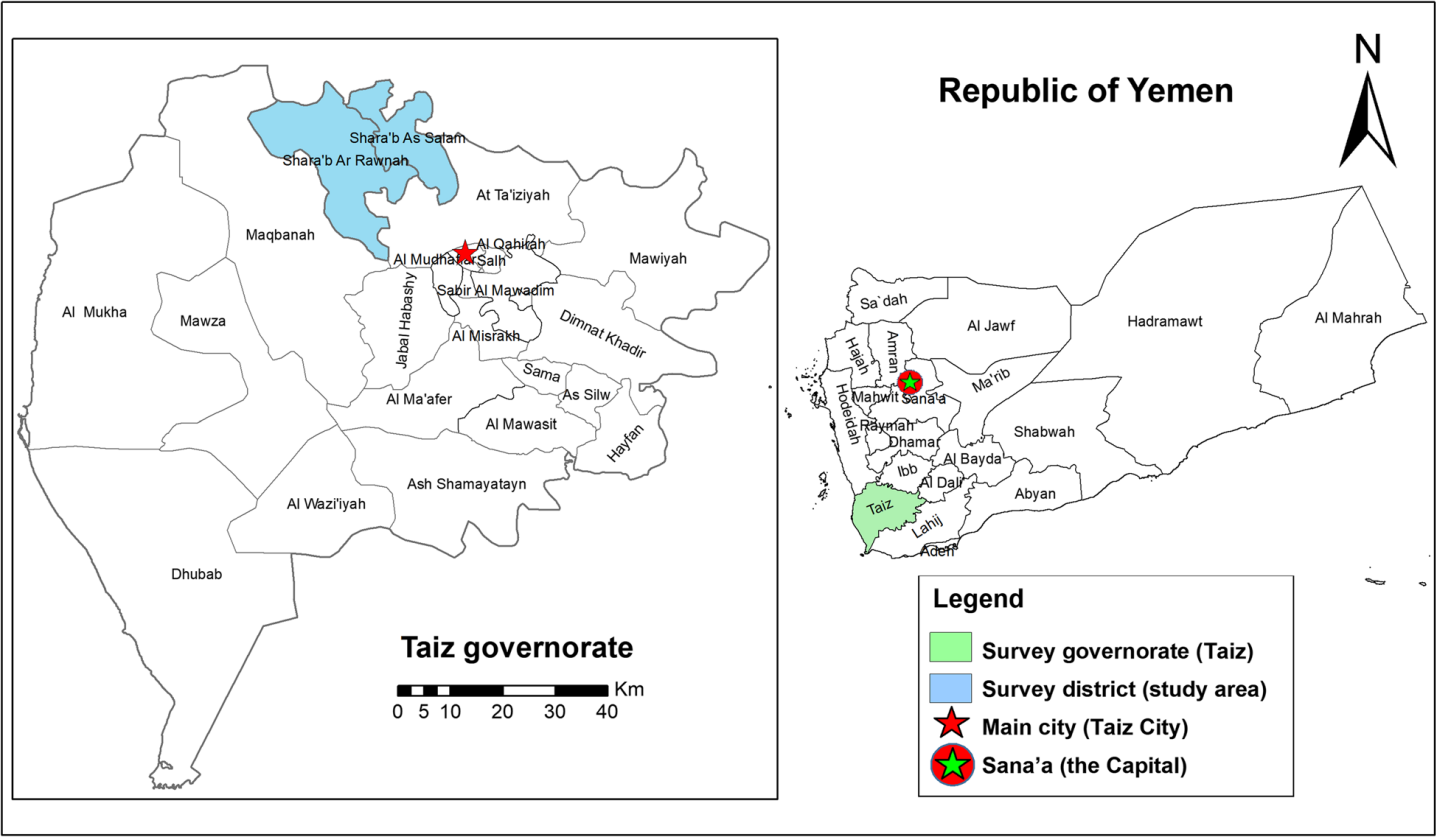
A geographic map showing the study area (Shara’b district) in Taiz governorate. The map was created by authors using the Esri ArcGIS 10.7 software

A cross-sectional survey-based study was carried out in five areas of Shara’b district in Taiz governorate, namely, Nakhla, Alamjood, Bani-Ziad, Bani-Sarry and Bani-Wahban (Fig. 1). Taiz governorate (44.01° E, 13.34° N) is located in the southwestern part of Yemen, 280 km from Sana’a, the capital. Shara’b district was selected because a recent study confirmed that it is endemic for CL [10]. The five abovementioned areas of Shara’b district were selected by simple random sampling from the 11 known CL-endemic areas identified by Asmaa et al. [10]. The administrative district of Shara’b is divided into two sub-districts: Shara’b As-Salam, which covers an area of 210 km^2^ and has a population of 146,650 and Shara’b ar-Rawnah, which encompasses an area of 417 km^2^ and is home to a population of 186,955. Shara’b district is mountainous with some valleys and lies at an altitude of approximately 2000 m above sea level. Hence it can be described as a highland district. A number of aqueducts can be found near villages in the district and water is permanently present in these aqueducts throughout the year. The climate in this highland district varies from arid to wet, with an annual rainfall of 600–800 mm. It is cold in winter and warm in summer, with a mean annual temperature of approximately 23 °C.

### Study population

Five hundred households were involved in this study. The household was selected as the sampling unit because decision making takes place at the household level. The five selected areas are almost homogeneous in terms of population size. Therefore, 100 households were selected from each area by simple random sampling using a list compiled by local administrators. During the research team visits to the study area, the head of each selected household, or another member if the head was not available, was invited to participate in this study. Verbal consent was obtained from each participant after they had been provided with an adequate explanation of the nature and objectives of this study. Only those who agreed to participate were interviewed. The study protocol was approved by the Medical Ethics Committee of the University of Taiz, Yemen. The principal investigator coordinated the interview process and also conducted daily spot-checks and reviews of the completed questionnaires to ensure the completeness and consistency of the collected data.

The minimum sample size required for this survey was calculated according to the WHO’s practical manual for sample size determination in health studies [14]. As previous data on the knowledge about and attitude towards CL in Yemen were unavailable, a 50% level of knowledge was considered, with a 95% confidence level and 5% level of significance. Thus, 384 was yielded as the minimum number of participants required for this study. However, to ensure that this sample size would be achieved, 500 households was targeted and used for the probability proportional to size sampling method in order to allow for non-participation and incomplete questionnaires.

The design, setting, analyses and reporting of this study adhered to the STROBE guidelines and criteria for cross-sectional studies in epidemiology [15] (see supplementary file 1).

### Data collection

Data collection was carried out over a period of 4 months from February to May 2019. A pretested structured questionnaire was utilised to collect data on the sociodemographic characteristics of the participants, their knowledge about and attitude towards CL and their knowledge on the sand fly vector. The questionnaire was developed based on a literature review of similar studies in other countries. In addition, some meetings were held with researchers, local healthcare staff and community members to identify the terms and questions that could be used for disease description. The questionnaire was designed in the English language and then translated into the Arabic language (the participants’ native language), and the local name (*Athrah*) for CL was used to refer to the disease.

The questionnaire was composed of four sections (see supplementary file 2). Section 1 consisted of questions designed to obtain data on the participants’ sociodemographic characteristics, such as age, gender, education level, occupation and number of household members. Section 2 contained questions on knowledge about CL that were developed to understand the participants’ ability to identify the disease, its signs and symptoms, its vector, as well as the peak period of incidence, treatment options, and prevention measures. Section 3 consisted of questions on knowledge about sand flies that were designed to determine the participants’ ability to identify and differentiate sand flies from other flying insects, as well as the breeding sites, time of biting and the disease(s) that can be transmitted by sand flies. Section 4 contained questions that were developed to gain an understanding of the participants’ attitudes towards CL in comparison to their attitudes towards malaria in terms of severity, prevention and curability. Malaria was selected as the comparison disease because the selected district is a malaria-endemic area and it is considered to be sufficiently well known among the public at large. This final section concluded with a question on the participants’ sources of information. Most of the questions were open-ended in order to avoid any false impressions due to guessing. However, the participants were requested to answer some questions by choosing one of three options: ‘no’, ‘yes’ or ‘I don’t know’. The participants were interviewed face to face in their household setting by three assistants who had received adequate training on the administration of the questionnaire and on the purpose of this study.

### Data analysis

Data were entered into Microsoft Office Excel spreadsheets and checked for accuracy and completeness by two research assistants. Data analysis was done using the Statistical Package for the Social Sciences version 18 (IBM Corp., New York, USA). Descriptive statistics such as frequency, percentage and mean were used to describe the knowledge and attitude components and the explanatory variables. The chi-square test was used to examine the association between the good knowledge scores and the explanatory variables such as age, gender, education level, occupation and household size. A multivariate logistic regression analysis was also performed to identify the significant determinants of good knowledge of CL and the sand fly vector, where a *P* level ≤ 0.25 in the univariate analysis was considered as the criterion for the inclusion of variables in the multiple logistic regression models [16]. Adjusted odd ratios (AORs) and their corresponding 95% confidence intervals (CIs) were calculated based on the final models. The significance level for all tests was set at *P* < 0.05.

Knowledge and attitude were scored according to the method described in [17], with some modifications to fit the studied disease. Each correct response to a question was assigned a score of 1 and each incorrect or unsure response was assigned a score of 0.

Knowledge about CL was assessed according to the responses given to five items. The correct answers to these five items were as follows: 1) a symptom of CL is a skin ulcer, skin wound or skin scar; 2) CL is transmitted through sand fly bites; 3) the peak of CL incidence is summer; 4) CL can be treated by herbal medicine, chemotherapy, or cauterisation; and 5) preventive measures include treating patients, vector control and improving awareness. Thus, the total knowledge score for CL ranged from 0 to 5. Knowledge scores between 0 and 3 were considered to indicate poor knowledge while scores of more than 3 were considered to denote good knowledge.

Knowledge about the sand fly vector was also assessed on the basis of five items. The correct responses to the five items were as follows: 1) the sand fly is the vector of CL; 2), the biting time is at night; 3) the breeding sites are holes in dry trees, and cattle and sheep dung; 4) the methods of control include using bed nets and insecticides and minimising close contact with animals by situating animal stables as far as possible from dwellings. As above, the total knowledge score for the sand fly vector ranged from 0 to 5 with a score of 0 to 3 indicating poor knowledge and a score above 3 indicating good knowledge.

Attitude towards CL was assessed based on the responses to three items. The correct answers were as follows: 1) CL is less dangerous than malaria; 2) CL can be treated; and 3) CL can be prevented. Each correct answer to an attitude items was given a score of 1. An attitude score of 0 or 1 was considered to denote a negative attitude while a score of 2 or 3 was considered to represent a positive attitude.

## Results

A total of 500 participants were recruited for this study, 34 of whom were excluded due to incomplete data. Hence, 466 participants were included in the final analysis.

### Sociodemographic characteristics

Of the 466 participants included in the final analysis, 62.7% were male and 37.3% were female. Most of the participants (65.7%) were aged between 18 and 40 years and approximately one third (34.3%) were older than 40 years. In respect of education level, 21.5% of the participants were non-educated while 39.7% had completed a secondary school level of education. Just over half (53.2%) of the participants were university students while 19.3, 14.8 and 7.5% were teachers, farmers and housewives, respectively. Most of the interviewed households (53.0%) were composed of five to nine members. Table 1 provides a full breakdown of the sociodemographic characteristics of the study participants.

**Table 1:** General sociodemographic characteristics of the study participants (*n* = 466)

Variables	Study location					Total n (%)
	Bani-Ziad n (%)	Bani-Sarry n (%)	Nakhla n (%)	Bani-Wahban n (%)	Al-Amjood n (%)	
No. of participants	79	94	94	99	100	466 (100)
Age (years)						
18–40	53 (67.1)	56 (59.6)	84 (89.4)	80 (80.8)	33 (33)	306 (65.7)
> 40	26 (32.9)	38 (40.4)	10 (10.6)	19 (19.2)	67 (67)	160 (34.3)
Gender						
Male	50 (63.3)	59 (62.8)	50 (53.2)	55 (55.6)	78 (78.0)	292 (62.7)
Female	29 (36.7)	35 (37.2)	44 (46.8)	44 (44.4)	22 (22.0)	174 (37.3)
Educational level						
Non educated	14 (17.7)	18 (19.1)	8 (8.5)	11 (11.1)	49 (49)	100 (21.5)
Primary	17 (21.5)	30 (31.9)	17 (18.1)	11 (11.1)	9 (9)	84 (18.0)
Secondary	33 (41.8)	21 (22.3)	63 (67)	46 (46.5)	22 (22)	185 (39.7)
Tertiary	15 (19.0)	25 (26.6)	6 (6.4)	31 (31.6)	20 (20)	97 (20.8)
Occupation						
Students	40 (50.6)	49 (52.1)	72 (76.6)	58 (58.6)	29 (29)	248 (53.2)
Employees	17 (21)	21 (22.3)	12 (12.8)	19 (19.2)	21 (21)	90 (19.3)
Housewives	6 (7.6)	11 (11.7)	5 (5.3)	2 (2)	11 (11)	35 (7.5)
Farmers	6 (7.6)	7 (7.4)	4 (4.3)	13 (13.1)	39 (39)	69 (14.8)
Not working	10 (12.7)	6 (6.4)	1 (1.1)	7 (7.1)	0 (0)	24 (5.2)
No. of household members						
< 5	18 (22.8)	22 (23.4)	13 (13.8)	23 (23.2)	11 (11)	87 (18.7)
5–9	49 (62.0)	41 (43.6)	34 (36.2)	49 (49.5)	74 (74)	247 (53.0)
> 9	12 (15.2)	31 (33.0)	47 (50.0)	27 (27.3)	15 (15)	132 (28.3)

### Knowledge about cutaneous leishmaniasis

Table 2 shows the results for the participants’ knowledge about the signs and symptoms, transmission, prevention and treatment of CL. From the table it can be seen that 76% (364/466) of the participants had seen a CL case before. When participants were asked about the signs and symptoms of CL, 39.5% (184/466) correctly answered that a skin ulcer is the main symptom of CL while 40.2% were either unable to mention any signs or symptoms of CL (25.3%) or mentioned non-specific symptoms (14.9%). With regard to mode of transmission, only 12.9% (60/466) knew that CL is transmitted by sand flies while 61.4% (286/466) were unable to mention any mode of CL transmission. Moreover, 25.8% of the participants demonstrated misconceptions about the transmission of CL, mentioning mosquitoes, autoinfection, and direct person-to-person skin contact. The results in the table also show that 35.2% of the participants correctly stated that the peak incidence of CL occurs in summer while 47.0% did not know the main transmission season.

**Table 2:** Knowledge about cutaneous leishmaniasis among the study participants (*n* = 466)

Variables	Response categories	n (%)
Have seen individuals infected with CL	Yes	354 (76.0)
	No	112 (24.0)
Signs and symptoms of CL	Skin ulcer	184 (39.5)
	Itching and redness	59 (12.7)
	Skin wound	51 (10.9)
	Skin scar	44 (9.4)
	Others	10 (2.1)
	I don’t know	118 (25.3)
Mode of transmission of CL	Sand fly biting	60 (12.9)
	Bites of mosquito & other flies	67 (14.4)
	Autoinfection	15 (3.2)
	Microorganisms	11 (2.4)
	Direct skin contact	27 (5.8)
	I don’t know	286 (61.4)
Period of CL incidence peak	Winter	79 (17.0)
	summer	164 (35.2)
	Autumn	2 (0.4)
	Spring	2 (0.4)
	I don’t know	219 (47.0)
Treatment options of CL	Herbal medicine	167 (35.8)
	Chemotherapy	92 (19.7)
	Cauterizing	10 (2.1)
	Self-heal	5 (1.1)
	Not treated	5 (1.1)
	Others	2 (0.4)
	I don’t know	185 (39.7)
Preventive measures of CL	Treating patients	93 (20.0)
	Eradicating vector	34 (7.3)
	Isolating patients	18 (3.9)
	Improving awareness	25 (5.4)
	Others	11 (2.4)
	I don’t know	285 (61.2)

As regards treating the disease, 35.8, 19.7 and 2.1% correctly stated that the methods of treatment for CL are herbal medicine, chemotherapy and cauterisation, respectively. As for prevention, the majority of the participants (62.9%) could not cite any preventive measure against CL while 20 and 7.3% correctly mentioned that treating infected patients and controlling the sand fly vector are preventive measures. Therefore, from the above results, the majority of the participants (77.7%) were found to have poor knowledge about CL (Table 3).

**Table 3:** Scores of knowledge and attitude towards cutaneous leishmaniasis and knowledge towards sand fly vector among the participants (*n* = 466)

Characteristics (Total score possible)	Knowledge and attitude scores (interpretation)	n	%
Knowledge towards CL (5)	0–3 (poor)	362	77.7
	4–5 (good)	104	22.3
Knowledge towards sand fly (5)	0–3 (poor)	312	67.0
	4–5 (good)	154	33.0
Attitude about CL (3)	0–1 (negative attitude)	231	49.6
	2–3 (positive attitude)	235	50.4

With regard to the participants’ sources of information, most (74.5%, 347/466) had heard about the disease through their families, relatives, and friends while 17.8% (83/466) had a personal history of infection. A further 6.2% (29/466) and 0.8% (4/466) had heard about CL at educational institutions and through social media, respectively.

### Knowledge about the sand fly vector

Table 4 contains the results for the participants’ knowledge about sand flies and their role in disease transmission. Interestingly, approximately half of the participants (49.1%) were able to identify and differentiate sand flies from other flies. However, only 14.8% (69/466) of the participants mentioned that CL is transmitted by sand flies. The majority of the participants (63.5%) did not know whether or not sand flies could transmit diseases, but 13.7% of the participants stated that sand flies can transmit malaria.

**Table 4:** Knowledge about sand fly vector among the participants (*n* = 466)

Variables	Response categories	n (%)
Can you identify and differentiate sand flies from other common flies and mosquitoes?	Yes	229 (49.1)
	No	237 (50.9)
Diseases transmitted by sand flies	Cutaneous leishmaniasis	69 (14.8)
	Malaria	64 (13.7)
	Itching and allergy	18 (3.9)
	Nothing	2 (0.4)
	Others	17 (3.6)
	I don’t know	296 (63.5)
Breeding places of sand flies	Swamps	53 (11.4)
	Sewers	19 (4.1)
	Cattle and sheep dung	78 (16.7)
	Dry tree holes	25 (5.4)
	Valleys	18 (3.9)
	Others	14 (3.0)
	I don’t know	259 (55.6)
Biting time of sand flies	From dusk to sunrise	101 (21.7)
	Day time	53 (11.4)
	Any time	29 (6.2)
	Others	8 (1.7)
	I don’t know	283 (60.7)
Methods to control sand flies	Using bed nets	106 (22.7)
	Using insecticides	48 (10.3)
	Situating animal stables as distant as possible from dwellings	12 (2.6)
	Personal hygiene	21 (4.5)
	Others	8 (1.7)
	I don’t know	271 (58.2)

With regard to breeding sites, 16.7% (78/466) and 5.4% (25/466) of the participants mentioned cattle and sheep dung, and holes in dry trees, respectively, but the majority (55.6%) did not know the answer. With respect to biting time, 21.7% of the participants correctly stated that sand flies bite during the night-time while 17.6% thought that sand flies bite at any time of the day. The majority of the participants (58.2%) were unable to mention any control measures against sand flies while 40.1% correctly answered that sand flies can be controlled by using bed nets, spraying insecticides, improving sanitation and avoiding breeding sites. Therefore, from the above results, it was found that about two-thirds of the respondents (67%) had poor knowledge about the sand fly acting as vector of CL (Table 3).

### Attitude towards cutaneous leishmaniasis

Table 5 gives the results for the participants’ attitude towards CL. A total of 205 (44%) participants considered CL to be a serious condition and more dangerous than malaria while 41.0% believed that CL is a mild infection. Interestingly, the majority of the participants (82.8%) showed a positive attitude and thought that the disease is curable while only 3.9% believed that the disease cannot be cured. However, 61.2% of the participants believed that CL cannot be prevented. From the analysis of these results, it is apparent that approximately half of the participants (49.6%) had a negative attitude towards CL (Table 3).

**Table 5:** Attitude towards cutaneous leishmaniasis among the participants (n = 466)

Variables	Response categories	n (%)
Is CL more dangerous than Malaria?	Yes	205 (44.0)
	No	191 (41.0)
	I don’t know	70 (15.0)
Is CL a curable disease?	Yes	386 (82.8)
	No	17 (3.6)
	I don’t know	63 (13.5)
Is CL a preventable disease?	Yes	170 (36.6)
	No	285 (61.2)
	I don’t know	11 (2.4)

### Factors associated with knowledge about and attitude towards CL and the sand fly vector

Table 6 shows that the percentage of participants aged over 40 years with good knowledge about CL is significantly higher than that of participants aged 18–40 years (28.1 vs. 19.3%; *P* = 0.029). Also, a significantly higher percentage of the participants with good knowledge about CL are male compared to female (25.3 vs. 17.2%; *P* = 0.042). The results in Table 6 also show that the percentage of participants with good knowledge about sand flies is significantly higher among those aged over 40 years and farmers as compared to younger and non-working participants, respectively (46.4 vs. 30.9%; *P* = 0.014). Also, the percentage of participants with good knowledge about sand flies is significantly lower among those with a primary level of education as compared to non-educated participants (22.6 vs. 40.0%; *P* = 0.022). On the other hand, the distribution of the scores for a positive attitude towards CL among the different groups is comparable (*P* > 0.05).

**Table 6:** Association of participants’ knowledge towards cutaneous leishmaniasis and sand fly vector with their sociodemographic factors

Variables	Good knowledge about CL^a^			Good knowledge about sand fly^a^		
	n (%)	COR (95% CI)	AOR (95% CI)	n (%)	COR (95% CI)	AOR (95% CI)
Age (years)						
> 40	45 (28.1)	1.64 (1.05, 2.56)^*^	2.40 (1.05, 5.50)^*^	64 (40.0)	1.60 (1.07, 2.39)^*^	1.51 (0.75, 3.04)
18–40	59 (19.3)	1	1	90 (29.4)	1	1
Gender						
Male	74 (25.3)	1.63 (1.02, 2.62)^*^	1.50 (0.88, 2.57)	88 (30.1)	0.71 (0.48, 1.05)	0.51 (0.32, 0.82)^*^
Female	30 (17.2)	1	1	66 (37.9)	1	1
Education						
Tertiary	17 (17.5)	0.61 (0.31, 1.20)	–	35 (36.1)	0.78 (0.44, 1.38)	1.41 (0.57, 3.51)
Secondary	39 (21.1)	0.76 (0.43, 1.34)	–	58 (31.4)	0.63 (0.38, 1.05)	0.71 (0.31, 1.60)
Primary	22 (26.2)	1.01 (0.52, 1.96)	–	19 (22.6)	0.40 (0.21, 0.77)^*^	0.43 (0.17, 1.08)
Non educated	26 (26.0)	1	–	42 (42.0)	1	1
Occupation						
Employees	21 (23.3)	1.18 (0.67, 2.07)	–	27 (30.0)	0.96 (0.57, 1.60)	0.52 (0.23, 1.21)
Farmers	20 (29.0)	1.58 (0.88, 2.85)	–	32 (46.4)	1.93 (1.13, 3.28)^*^	1.39 (0.66, 2.93)
Not working (students & housewives)	63 (20.5)	1	–	95 (30.9)	1	1
No. of household members						
> 9	28 (21.2)	0.96 (0.50, 1.86)	–	41 (31.1)	0.81 (0.46, 1.44)	–
5–9	57 (23.1)	1.07 (0.60, 1.93)	–	82 (33.2)	0.90 (0.54, 1.50)	–
< 5	19 (21.8)	1	–	31 (35.6)	1	–

All values are number (%). *COR* CRUDE ODDS ratio, *AOR* adjusted odds ratio, *CI* confidence interval

^*^ Significant association (*P* < 0.05)

^a^Based on scores shown in Table 3

The outcome of the multivariate logistic regression presented in Table 6 shows that age and gender were significant determinants of good knowledge about CL and sand flies, respectively. The participants aged over 40 years were about two times more likely to have good knowledge about CL as compared to the younger participants (AOR = 2.40; 95% CI = 1.05, 5.50). Interestingly, male participants were 0.71 times less likely to have good knowledge about sand flies compared to their female counterparts (AOR = 0.51; 95% CI = 0.32, 0.82). The other sociodemographic variables (occupation and education level) were not retained in the multivariate analysis.

## Discussion

The current study investigated knowledge about and attitude towards CL in five rural CL-endemic communities in Shara’b district, southwestern Yemen. The findings indicate that CL is familiar to the targeted community. The study participants described a CL lesion using the following terms: *Athrah* (scar), *Shiqna* (lesion that affects all family members), *Nafta* (nodular lesion) or *Bula* (wet lesion). Local vernacular names differ from region to region and are mostly related to the lesion morphology, the aesthetic and social stigmata associated with a disease, and the disease course [18]. Notably, the participants in the current study considered a skin lesion on the face to be a symptom of CL.

The majority (76.0%) of the participants had seen CL cases within the community either among family members or other persons in the locality. This result seems to be a direct consequence of the high endemicity of CL in the investigated areas that causes the population to be aware of the signs and symptoms of the disease. Unfortunately, the lack of previous investigations on the level of knowledge about CL and/or CL-related stigma among the Yemeni population prevents a comparison of the results of the current study with other prior findings. However, this result is aligned with that reported for the Volta region in Ghana (an endemic area) where 82.0% of participants stated that they had seen CL cases and that skin lesion is the main symptom [19]. In contrast, a knowledge, attitude and practice (KAP) study carried out in Alexandria, Egypt (a non-endemic area) found that the majority of the participants (90%) had never seen an infected person [20].

Although most of the participants in the current study had knowledge of the symptoms of CL, unexpectedly they had poor knowledge about the mode of disease transmission: only 12.9% of the participants knew that the sand fly is the vector for CL. These findings are consistent with a previous study conducted in endemic areas in Saudi Arabia [21]. The findings of the current study also show that a sizeable proportion of the participants (25.8%) had misconceptions about the mode of transmission, some citing housefly bites and autoinfection as possible causes. This finding is in agreement with a study conducted in the Hail region in Saudi Arabia, where the majority of the participants exhibited misunderstandings about the transmission of CL [22]. In contrast, a better level of knowledge about CL transmission has been reported by studies undertaken in Nepal, Brazil and Iran [17, 23–25]. This variation in the knowledge level between countries could be related to sociocultural factors. However, it is also important to point out that the transmission cycle of *Leishmania* has particular features that differ from one endemic area to another according to the geoclimatic conditions of the study context. Therefore, the extrapolation of the results from one region to another is not recommended.

In the current study, the majority of the participants believed that CL is curable and about one third of the participants thought that CL can be treated by herbal medicine and mentioned some traditional plants that are used to cure CL. Worryingly, some participants recommended the application of harmful acids on lesions as a treatment for CL. These findings show that there was poor knowledge among the local community regarding the usage of modern treatment strategies. This low level of knowledge could be a consequence of the collapse of the public health system in Yemen due to the ongoing armed conflict [13]. Knowledge about personal preventive measures such as wearing long-sleeved clothing, using mesh over windows, and using bed nets or repellents was also poor. In contrast, KAP surveys conducted in Pakistan and Syria have revealed that local communities are aware of the preventive measures they need to take against CL [26, 27]. This knowledge was acquired from governmental mass media campaigns about vector-borne diseases including dengue, malaria and CL. In contrast, in Yemen, the only control programme, which solely targets malaria, is largely paralysed due to the civil war.

With regard to attitude towards CL, the majority of the participants in the current study believed that CL is a serious disease and that it is more dangerous than malaria. This attitude could most likely be a consequence of the high endemicity of CL in the study area and the chronicity of the associated lesions that result in disfiguring scars. Such scars lead to serious psychological and social suffering including stigma, social exclusion and mental distress [4]. Overall, half of the participants involved in the current study had a negative attitude towards CL. This could be a direct consequence of a lack of access to information about CL. Unfortunately, a negative attitude may lead to a delay in seeking treatment and, in the case of CL, may in turn lead to complications such as deep tissue damage, secondary infections, mutilating scars and negative psychological impact.

Most of the participants in the current study were able to identify sand flies. Locally, the sand fly is called *Hass* (painful-biting dipteran) and *Katem Sout* (silent dipteran). The ability of the participants to identify this dipteran may be due to many of them living in close proximity to domestic animals and in poor housing conditions both of which are suitable environments for the breeding of sand flies. However, although the participants were able to identify and differentiate sand flies from other flies, a significant proportion of the participants did not know about the role played by the phlebotomine sand fly in the transmission of CL. Also, the majority of the participants did not have correct knowledge about the peak season of transmission, the locations of sand fly breeding sites, the biting time or control methods. Conversely, a previous study in Isfahan, Iran, which reported that 89.8% of the participants knew about the role of the sand fly as a vector for CL, but only 13.9% had enough information on the criteria by which to differentiate sand flies from other flies [28].

Overall, the findings of the current study reveal that the rural community in Taiz governorate had poor knowledge about and attitude towards CL and its sand fly vector. However, interestingly, participants aged over 40 years had a better level of knowledge about CL compared to those aged 18–40 years. This difference could be attributed to the gaining of increased experience over time in respect of personal history of infection and/or seeing other people infected with CL.

The findings also indicate that the male participants had better knowledge about CL as compared to their female counterparts, which could be explained by the fact that the male population is more likely to be infected with CL in the study area [10] and in the northwestern region [7]. However, the association between gender and knowledge about CL was not retained in the multivariate analysis. On the other hand, the findings also show that the female participants had a better level of knowledge about the sand fly vector as compared to their male counterparts. In rural Yemen, including the study area, humans live in close contact with animals because the ground floor of dwellings is traditionally occupied by animals, especially cows, and some households also have space for sheep within or near the dwelling, and it is such conditions that provide favourable breeding sites for sand flies. The female members of the household are primarily responsible for animal husbandry activities. Therefore, it would follow that they would be more familiar with the presence of sand flies as compared to the male family members. Similarly, although the occupation variable was not retained in the multivariate analysis, farmers were also found to have significantly better knowledge about sand flies as compared to students and employees. However, farmers did not know about the role of sand flies in the transmission of CL.

The overall poor level of knowledge of CL revealed by the current study could most likely be a direct consequence of this disease being neglected by health policymakers and public health professionals and a lack of priority being given to the implementation of control measures. The situation also became more complicated in 2010 due to the Arab Spring movement and the ensuing political crisis. Unfortunately, in addition to this, the ongoing civil war in Yemen has resulted in the re-emergence and outbreak of several infectious diseases including dengue [29], cholera [30] and diphtheria [31], which is putting pressure on limited resources. Indeed, Taiz, the area of interest in this study, is one of the governorates experiencing continuous armed confrontations. It is strongly affected by the civil war that has led to the total collapse of the health system in the governorate.

In light of the above, the current study is timely in that it is the first to provide detailed information on the knowledge about and attitude towards CL and its vector among the Yemeni population, specifically in Taiz. However, it should be noted that this study has some limitations that should be considered when interpreting the findings. First, because this study was cross-sectional rather than interventional, this does not allow causal inference. Second, the lack of previous investigations on knowledge and attitude towards CL and CL-related stigma in Yemen prevented the comparison and evaluation of the findings with those of other relevant studies. Third, it was not possible to obtain information on the participants’ practices in relation to CL. Although the questionnaire was designed and validated to collect relevant information on KAP, the items on risk behaviour in relation to CL, family income, housing conditions and household assets were omitted due to the refusal of participants to provide this information. This was due to unforeseen tribal tensions and unrest during the study period. Fourth, qualitative questions might have helped to better elucidate the attitudes towards CL held by the participants. However, such questions were not included in the study. It is considered that including such items and questions would have revealed a more comprehensive picture. Nevertheless, the findings of this study might be generalisable to all of the rural communities in the Taiz governorate that are known to be endemic for CL. However, further studies may be required to confirm this conjecture.

## Conclusions

The current study revealed that within the rural community in Taiz, Yemen there is a poor level of knowledge about the cause, transmission, treatment and preventive measures for CL and a poor level of knowledge about the sand fly as a vector for CL. These findings would seem to be a direct consequence of a lack of attention being paid to this disease and a lack of priority being given to control measures; a situation that has been exacerbated since the onset of civil war in Yemen and the consequent breakdown of the healthcare infrastructure. In light of the findings of this study, there is an urgent need for health education to improve awareness and correct misconceptions about all aspects of CL in order to reduce incidence and prevalence in endemic areas.

## Supplementary Information

**Additional file 1.** STROBE checklist of the study.

**Additional file 2.** Questionnaire used to assess participants’ knowledge and attitude towards CL.

## Data Availability

The datasets used and/or analysed during the current study are available from the corresponding author on reasonable request.

## Abbreviations

CL: Cutaneous leishmaniasis
AOR: Adjusted odds ratio
CI: Confidence interval
WHO: World Health Organization
