# Supplementary material for: Knowledge and attitude towards cutaneous leishmaniasis among rural endemic communities in Shara’b district, Taiz, southwestern Yemen
Source: BMC Infect Dis. 2021 Mar 17;21:269. doi: 10.1186/s12879-021-05965-4 (PMC7968254; doi:10.1186/s12879-021-05965-4)
Supplement: Supplementary file 2 — Additional file 2. Questionnaire used to assess participants’ knowledge and attitude towards CL. [file 12879_2021_5965_MOESM2_ESM.pdf]

QUESTIONNAIRE

A community-based study on the knowledge and attitude towards cutaneous leishmaniasis among rural communities in endemic areas in Taiz

Reference No.

1. PERSONAL INFORMATION

Name (optional): …………………………………………..

Age: …………… years

Sex: Male ☐ Female ☐

Address (village name): …………………………………………..

Telephone no. (optional): …………………………………………..

Education: Never go to school ☐ Primary ☐ Secondary ☐ Tertiary ☐

Occupation: Government employee ☐ Farmer ☐ Student ☐ Housewife ☐ Not working ☐ Other: ……………………………………

No. of family members in the same household: ……………………

2. KNOWLEDGE ABOUT CUTANEOUS LEISHMANIASIS

Have you heard about cutaneous leishmaniasis before this time? Yes ☐ No ☐

If Yes, what is CL? …………………………………………………………

Have you ever got CL? Yes ☐ No ☐

If yes,

- Since when? ………………………………………………
- How many lesions scar: ………………………………………………
- Site of lesions: ………………………………………………

How can we know if someone has CL? ………………………………………………

Have you ever seen individuals infected with CL? Yes ☐ No ☐

What are the signs and symptoms you know related to CL?

1- …………………. 2- …………………..
3- …………………. 4- …………………..
5- …………………. 6- …………………..
Don't know ☐

How CL can be transmitted? ………………………………………………
Don't know ☐

In which season is CL more common?
Summer ☐ Autumn ☐
Winter ☐ Spring ☐
Don't know ☐

If you get CL, what do you do? ………………………………………………

How CL can be treated?
1- ……………………………………………
2- ……………………………………………..
3- ……………………………………………….
Don't know ☐

How CL can be prevented?
1- ……………………………………………
2- ……………………………………………..
3- ……………………………………………….
4- ……………………………………………..
Don't know ☐

Do you have mosquito bed nets at your household? Yes ☐ No ☐

3. KNOWLEDGE ABOUT CL VECTOR

Have you ever seen sand flies? Yes ☐ No ☐

If yes,

- Have you observed sand flies at your household area? Yes ☐ No ☐
- During what hours of the day? ……………………………………………..

In what places sand flies can be commonly found?	1- ……………………………………………<br>2- ……………………………………………<br>3- ……………………………………………<br>Don't know ☐
Can you distinguish sand flies from other flies and mosquitoes?	Yes ☐ No ☐
If yes; please tell me how?	……………………………………………<br>……………………………………………
What are the diseases transmitted by sand flies?	1- ……………………………………………<br>2- ……………………………………………<br>3- ……………………………………………<br>Don't know ☐
During what hours of the day sand flies can bite individuals?	Morning ☐ Night ☐<br>Day time ☐ Any time ☐<br>From dusk to sunrise ☐ Don't know ☐<br>Other: …………………………………
How the sand flies can be controlled?	1- ……………………………………………<br>2- ……………………………………………<br>3- ……………………………………………<br>4- ……………………………………………<br>Don't know ☐

4. ATTITUDE TOWARDS CUTANEOUS LEISHMANIASIS

Is CL a serious disease?	Yes ☐ No ☐ Don't know ☐
Is CL more dangerous than Malaria?	Yes ☐ No ☐ Don't know ☐
Is CL curable?	Yes ☐ No ☐ Don't know ☐
Is CL preventable?	Yes ☐ No ☐ Don't know ☐
Could CL be transmitted by direct contact from person to person?	Yes ☐ No ☐ Don't know ☐
From where you came to know about CL?	Family ☐ Friends ☐<br>School ☐ Media ☐<br>History of infection ☐<br>Other: ……………………………………………<br>Don't remember ☐

-Thank you for your kind cooperation-
